# Chronic Gastrointestinal Disorders and miRNA-Associated Disease: An Up-to-Date

**DOI:** 10.3390/ijms26010413

**Published:** 2025-01-06

**Authors:** Alessandro Giammona, Bruno Giovanni Galuzzi, Elena Imperia, Clarissa Gervasoni, Sofia Remedia, Laura Restaneo, Martina Nespoli, Laura De Gara, Flaminia Tani, Michele Cicala, Michele Pier Luca Guarino, Danilo Porro, Antonio Cerasa, Alessia Lo Dico, Annamaria Altomare, Gloria Bertoli

**Affiliations:** 1 Istituto di Bioimmagini e Sistemi Biologici Complessi (IBSBC), National Research Council (CNR), Segrate, 20054 Milan, Italy; alessandro.giammona@cnr.it (A.G.); brunogiovanni.galuzzi@cnr.it (B.G.G.); clarissa.gervasoni@cnr.it (C.G.); sofiaremedia@cnr.it (S.R.); martinanespoli@cnr.it (M.N.); flaminiatani@cnr.it (F.T.); danilo.porro@cnr.it (D.P.); antonio.cerasa@cnr.it (A.C.); 2 National Biodiversity Future Center (NBFC), 90133 Palermo, Italy; 3 Department of Sciences and Technologies for Sustainable Development and One Health, Università Campus Bio-Medico di Roma, Via Alvaro del Portillo 21, 00128 Rome, Italy; elena.imperia@unicampus.it (E.I.); laura.restaneo3@gmail.com (L.R.); l.degara@unicampus.it (L.D.G.); a.altomare@policlinicocampus.it (A.A.); 4 Dipartimento di Scienze della Terra e del Mare (DISTEM), Università di Palermo, Via Archirafi, 22, 90123 Palermo, Italy; 5 Research Unit of Gastroenterology, Università Campus Bio-Medico di Roma, Via Alvaro del Portillo 21, 00128 Rome, Italy; m.cicala@policlinicocampus.it (M.C.); m.guarino@policlinicocampus.it (M.P.L.G.); 6 Unit of Gastroenterology, Fondazione Policlinico Campus Bio-Medico di Roma, Via Alvaro del Portillo 200, 00128 Rome, Italy; 7 Dipartimento di Biotecnologie e Bioscienze, Università degli Studi di Milano Bicocca, 20126 Milan, Italy

**Keywords:** inflammatory bowel disease, microRNA, hallmarks of IBD/IBS, inflammation, oxidative stress, apoptosis, colorectal cancer

## Abstract

Chronic gastrointestinal disorders such as inflammatory bowel diseases (IBDs) and irritable bowel syndrome (IBS) impose significant health burdens globally. IBDs, encompassing Crohn’s disease and ulcerative colitis, are multifactorial disorders characterized by chronic inflammation of the gastrointestinal tract. On the other hand, IBS is one of the principal gastrointestinal tract functional disorders and is characterized by abdominal pain and altered bowel habits. Although the precise etiopathogenesis of these disorders remains unclear, mounting evidence suggests that non-coding RNA molecules play crucial roles in regulating gene expression associated with inflammation, apoptosis, oxidative stress, and tissue permeability, thus influencing disease progression. miRNAs have emerged as possible reliable biomarkers, as they can be analyzed in the biological fluids of patients at a low cost. This review explores the roles of miRNAs in IBDs and IBS, focusing on their involvement in the control of disease hallmarks. By an extensive literature review and employing bioinformatics tools, we identified the miRNAs frequently studied concerning these diseases. Ultimately, specific miRNAs could be proposed as diagnostic biomarkers for IBDs and IBS. Their ability to be secreted into biofluids makes them promising candidates for non-invasive diagnostic tools. Therefore, understanding molecular mechanisms through the ways in which they regulate gastrointestinal inflammation and immune responses could provide new insights into the pathogenesis of IBDs and IBS and open avenues for miRNA-based therapeutic interventions.

## 1. Introduction

Over the past decade, significant progress has been made in understanding the pathophysiology of Crohn’s disease (CD) and ulcerative colitis (UC)—the two primary forms of inflammatory bowel disease (IBD)—as well as irritable bowel syndrome (IBS) [1,2]. IBD represents one of the most debilitating conditions in gastroenterology, associated with considerable morbidity and, in some cases, mortality. Typically present during adolescence or early adulthood, IBD can result in chronic suffering and severely impact patients’ quality of life. Additionally, it imposes substantial economic burdens due to frequent medical care and work absenteeism [3]. By contrast, IBS is a functional gastrointestinal disorder affecting 5–10% of the global population and does not directly influence mortality. However, its pervasive symptoms significantly diminish patients’ quality of life, underscoring its importance in gastroenterology [2].

Genetic studies suggest that the phenotypes of IBD, and potentially IBS, likely represent the final outcome of numerous distinct disease processes [4,5]. The strongest genetic associations have been reported for CD, with very convincing evidence for Card15/NOD-2 and the autophagy genes ATG16L1 and IRGM, all associated with the processing of microbial antigens and innate immunity [6]. However, it needs to be emphasized that, while genetic susceptibility is of crucial importance, the penetrance of these disorders is environmental [7]. In this context, growing evidence suggests that intestinal microbiota plays a crucial role in the development of IBD and IBS [8,9]. Changes in the abundance of specific bacterial species have been observed in IBD, and research using murine models of colitis indicates that gut microbiota can either exacerbate or mitigate ongoing mucosal inflammation [8]. Despite the heterogeneity of the studies, in IBS patients’ specific intestinal microbiota, alterations were also identified with a predominant presence of Firmicutes and Actinobacteria in fecal microbiota, with a higher abundance of Ruminococcaceae [10]. Microbiota composition alterations impair intestinal cellular metabolism, resulting in the modulation of several molecules involved in both inflammation and oxidative stress [11].

The use of specific clinical biomarkers for a differential diagnosis of IBD and IBS is still a challenge. From a molecular point of view, IBD and IBS diagnoses are mainly based on the specific methodology described below, associating clinical symptom observations with family history and the quantification of fecal calprotectin or lactoferrin and C-reactive protein, an overall inflammation biomarker [12]. Otherwise, routine colonoscopy is recommended in patients suspected of having IBD or IBS, and this has a high impact on patient management, as well as high medical costs.

Much evidence has highlighted that IBDs are characterized by a strong unphysiological inflammatory reaction involving the complex deregulation of many genes. This could also be relevant in IBS because recent studies have shown that the colons of patients with IBS are infiltrated by inflammatory cells [12].

The altered inflammatory pathways on these pathologies would be regulated by microRNAs (miRNAs) [13,14,15]. MiRNAs are small, single-stranded, non-coding RNA, able to target specific protein-coding mRNAs in several cellular pathways, as reported previously [16,17]. Specific miRNAs emerge as critical players in IBD and IBS diseases by regulating some key inflammatory, proliferative metabolic homeostasis, apoptosis, and many other genes. The deregulation of specific miRNAs could have an important impact on the development of the clinical alterations observed in these pathologies.

Therefore, these small molecules have garnered growing interest for their regulatory role in cell function and potential involvement in the onset and progression of IBD and IBS. Their presence in biofluids like urine, saliva, and blood suggests they could serve as reliable, cost-effective biomarkers for these conditions [13,14,15,18].

Several factors could influence the onset of these two pathologies; in the view of clarifying the role of each of these factors, we summarized the main clinical characteristics of IBD and IBS, describing the impact of genetics, epigenetics, and the environment on the diseases’ onset, also highlighting the role of biodiversity loss on human health [19]. After defining the two pathologies from a clinical point of view, we exploited bioinformatics research to scan all the crucial literature documents and generate an extensive list of miRNAs that regulate the hallmark processes of IBD/IBS diseases. Specifically, we focused on recent papers from the last five years that discuss these pathologies and report clear miRNA involvement. As a result, we generated a collected, detailed view of miRNAs controlling biological pathways that have a major role in IBD (CD and UC) or IBS.

## 2. Chronic Gastrointestinal Disorders

### 2.1. IBDs: Definition

Inflammatory bowel diseases are a group of autoimmune disorders primarily affecting the gastrointestinal tract. Crohn’s disease (CD) is distinguished by discontinuous, or patchy, inflammation that can manifest anywhere along the digestive tract, from the mouth to the anus, involving multiple layers of the intestinal wall. The sites most frequently affected include the small intestine, particularly the terminal ileum, and the colon. Ulcerative colitis (UC) is a chronic inflammatory condition that affects the colon, specifically targeting the mucosal layer, and it is characterized by a neutrophilic infiltrate within the intestinal crypts and the lamina propria [20]. Consequently, inflammation and ulceration are predominantly limited to the epithelial layer, with occasional involvement of the submucosal layer, particularly in the large intestine, including the colon and rectum [1] (Table 1).

CD can manifest at any age but predominantly affects young adults, with approximately 25% of patients with IBD being under the age of 20. In the United States and Canada, the disease is diagnosed in at least four individuals per 100,000 live births, and its incidence and prevalence are rising globally, particularly in developing countries. By contrast, the peak onset of ulcerative colitis (UC) typically occurs during early adulthood [3,21].

The diagnosis relies on a comprehensive evaluation of clinical, radiological, endoscopic, and histopathological findings. However, a standardized diagnostic test is currently unavailable [1].

The gold standard for assessing CD activity is the endoscopic scoring systems evaluation. These metrics are fundamental for evaluating the efficacy and safety of drugs aimed at inducing and maintaining remission and achieving mucosal healing. Moreover, abdominal computed tomography (CT) is the most widely recommended first-line radiological study to evaluate active CD with suspected complications [22]. In recent years, MRI colonography with a higher diagnostic accuracy is used to evaluate the disease, avoiding passive exposure to ionizing radiation in young populations [22].

There are several multi-pronged scoring systems to measure the clinical severity of IBDs, but the most widely used are the CD Activity Index (CDAI) for CD and, the Harvey–Bradshaw Index (HBI) score for UC [22]. By contrast, colonic unclassified IBD as CD or UC (approximately 15%) cases are labeled “indeterminate colitis” (IC) due to undefined clarification criteria for CD and UC [1]. Furthermore, another 15% of colonic IBD cases undergoing pouch surgery, i.e., restorative proctocolectomy with ileal pouch–anal anastomosis (RPC-IPAA) for a definitive diagnosis of UC based on the final designation of endoscopic biopsies by the pathologist will have an initial diagnosis [23]. Moreover, the Mayo System score can be used to describe the severity of the disease and monitor patients during therapy [24]. These guidelines are valid for patients admitted to hospital with the following Truelove and Witts criteria: six or more movements of hematochezia (bloody diarrhea) per day with at least one marker of inseparable toxicity, including heart rate/rate > 90 beats/min, body temperature > 37.8 °C, blood hemoglobin < 10.5 g/dL, and/or an erythrocyte sedimentation rate (ESR) of −30 mm/h [25]. The most important clinical and pathological characteristics of IBDs are described in Table 1.

#### 2.1.1. IBD: Etiopathogenetic Mechanisms

The exact cause of inflammatory bowel disease (IBD) is not well understood, but it is believed to stem from a dysregulated immune response affecting the intestinal wall in genetically predisposed individuals. Those with IBD often show altered intestinal microbiota, although it remains uncertain whether this dysbiosis is a cause or a consequence of the disease. A significant risk factor for developing IBD is having a family history of the condition, with a stronger genetic association linked to childhood-onset compared to adult-onset IBD [3].

**Figure 1:**
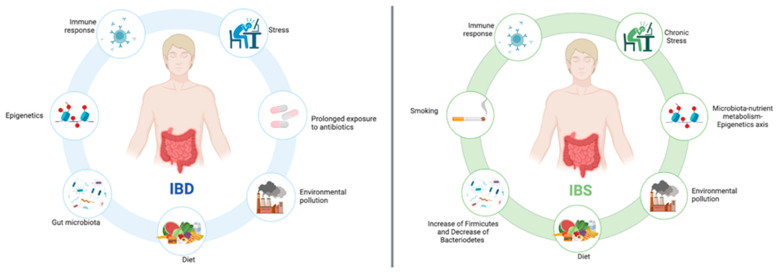
Principal etiopathogenetic factors involved in IBD and IBS, including external exposome factors (diet, environmental pollution, smoking, etc.), specific external exposome factors (exposure to antibiotics, gut microbiota composition, stress) and internal exposome factors (immune response, epigenetics, etc.).

IBD pathophysiology is multifactorial and belongs to interactions between genetics, environment, microbes, the immunological system, and potentially other risk factors, as summarized in Figure 1 [8]. In this paragraph, the following risk factors will mainly be described: epigenetic alterations, the interaction between diet and intestinal microbiota, and other significant environmental factors.

#### 2.1.2. Epigenetic Alterations

Epigenetic alterations have been reported in the context of IBD. Epigenetic changes result in alterations in gene expression through changes in DNA hypermethylation or hypomethylation, histone modification, and chromatin remodeling, without changing the DNA sequence, typically occurring at sites of cytosine followed by guanine (CpG). Epigenetic modifications, persisting throughout cycles of cell division and replication, can be inherited [8]. Numerous external exposome factors, such as environmental harm (pollution, smoking, heavy metals, and inorganic chemicals) has been linked to epigenetic alterations, especially when they occur early in life [8,26]. An early warning System for Cultural Heritage (EWAS) analysis of DNA from whole blood samples of 240 newly diagnosed patients with IBD (121 with CD and 119 with UC) and 191 individuals as controls (74 symptomatic without IBD and 117 healthy individuals) revealed 439 differentially methylated positions and 5 differentially methylated regions, and these results were replicable in an independent cohort [4].

Over 250 loci are affected by IBD risk, but far fewer are mechanistically linked to IBD, such as variants in NOD2, IL-23R, and 1ATG16L1, which are involved in the innate immune response against bacterial antigens, host–microorganism interactions, and autophagy [8]. Interestingly, technological innovation and the growing availability of cost-effective microarrays to assess genome-wide methylation has led to extensive progress in the field of epigenetic studies in complex diseases [7]. In detail, a mutation of the NOD2/CARD15 gene determines the alteration in intestinal immune homeostasis and the components that maintain the mucus layer [27]. Among Caucasians, loss-of-function mutations in the NOD2/CARD15 gene can be identified in 10% to 30% of patients with CD [7]. This gene, expressed in Paneth epithelial cells, neutrophils, and macrophages, is implicated in the recognition of the muramyl dipeptide of the peptidoglycan of the bacterial cell wall, activating the NF-kB pathway [28]. Most people with the NOD2/CARD15 gene mutation do not develop IBDs; this mutation confers a 3-fold increased risk of developing CD among heterozygotes and a 20-fold increased risk for homozygotes with a penetrance of approximately 5% [3]. Regarding the ATG16L1 gene, it alters the function of Paneth cells, reducing the protection against infections [27]. Genome-wide association studies (GWAS) suggest 20 other SNPs linked to loci associated with Th17-regulating intracellular networks and signal transduction, indicating the important role of Th17 in the pathogenesis of IBD, including IL-12B, JAK2, STAT3, and CCR6 [29].

#### 2.1.3. The Interaction Between Diet and Intestinal Microbiota

The interaction between diet and intestinal microbiota is a critical and consistently highlighted factor in inflammatory bowel disease (IBD) research [30,31]. Nutritional patterns have a profound impact on the composition of the gut microbiome and, consequently, on the immune response within the gut. A key factor in the pathogenesis of IBD is the pre-illness diet [30]. The Western-style diet (WSD) differs greatly from the traditional diet of previous generations due to a shift from a plant-based diet to a predominantly animal-based diet. This dietary pattern is characterized by high-calorie foods rich in carbohydrates, saturated fats, and animal proteins, alongside a marked reduction in fruits, vegetables, legumes, raw foods, and dietary fiber when compared to traditional diets such as the Mediterranean diet [30]. The WSD is also distinguished by its elevated levels of omega-6 fatty acids, primarily sourced from beef and pork, and a reduced intake of omega-3 polyunsaturated fatty acids (PUFAs) found in fish, resulting in a high omega-6 to omega-3 ratio [30]. Polyunsaturated fatty acids play a pivotal role in IBD pathogenesis by modulating immune responses and promoting intestinal inflammation. As precursors to eicosanoids, long-chain PUFAs are essential in the production of leukotrienes and prostaglandins, which are key mediators of inflammation [30].

By contrast, in patients with IBD in the EPIC cohort, a high intake of omega-3 fatty acids was related to a reduction in the risk of developing UC (*p* = 0.03) [32]. This is one of the many reasons why the Western diet has been considered a critical factor in creating intestinal inflammation [30]. Alterations in the composition and function of the intestinal microbiota, defined as dysbiosis, could be an important pathogenetic factor for IBD. Dysbiosis in patients with IBD can impair epithelial barrier function, resulting in defective bacterial recognition [19].

In recent years, substantial evidence has emerged concerning the role of gut microbiota in the immunopathogenesis of IBDs. The intestinal microbiota plays an important role since it can be modified through its interactions with environmental factors or by the genetic predisposition of the host [20,26]. The Western-style diet has been associated with markedly reduced microbiome diversity, a reduced abundance of *Prevotella*, and a greater number of *Bacteroides* spp. compared to that of Eastern populations [30], while a diet composed primarily of plant-based foods is related to a greater abundance of *Roseburia*, *Lachnospira*, and *Prevotella*, as well as an increased production of short-chain fatty acids (SCFAs) [30]. While the fermentation of simple carbohydrates produces SCFAs that maintain a healthy intestine, the fermentation of protein residues produces metabolites such as organic acids, phenolic compounds, indoles, and ammonia that are harmful and toxic to the intestine [30].

#### 2.1.4. Environmental Factors

The geographic distribution of IBD suggests a significant role of environmental factors in their pathogenesis. Historically, the disease first emerged in the northernmost regions of Europe and North America, gradually spreading southward over the following century. While the distinct north–south gradient in the Northern Hemisphere persists, it has diminished over time. Migration studies further highlight the influence of environmental factors. Individuals who move from regions with a high incidence of IBD to areas with lower incidence exhibit a reduced risk of developing these conditions, with the risk decreasing the younger they are at the time of migration. However, these findings are not universal. For example, individuals migrating from South Asia to Canada or England are at a higher risk of developing IBD compared to those migrating from China to Canada. This disparity suggests that additional factors, beyond environmental exposure, may mitigate or exacerbate the risk of developing these chronic conditions [3].

#### 2.1.5. Air Pollution

Air pollution could also play a role in the development of IBD [8,26]. Prenatal and early childhood exposure to air pollution, including polycyclic aromatic hydrocarbons (PAHs) and fine particulate matter (PM_2.5_), has been associated with childhood obesity and cardiometabolic health using traffic exposure data of road and residential pollution near the roadway [33,34,35]. The protective effects of green space, a measure of natural vegetation, on atopic sensitization have been studied in children [36,37], but such studies have been limited in the context of IBD. Exposure to reactive oxygen species during childhood was associated with an increased risk of IBDs (*p* < 0.05), while associations with other air pollutants were not statistically significant [38]. Furthermore, green space during early childhood has also been found to be protective against late-onset IBD in a dose-dependent manner [38].

Tobacco smoking is not robustly related to IBD risk, with a mild 13% risk increase for CD and none for UC [39]. The most consistently reported potential risk factor for CD is tobacco smoking, showing risk increases higher than 50%. By contrast, the role of smoking on UC has been observed as protective with similar strength [39].

### 2.2. IBS Clinical Definition

IBS is a chronic functional gastrointestinal disorder that impacts the brain–gut axis, and, as defined by the latest Roma IV criteria published in 2016, it manifests through symptoms such as abdominal pain and changes in bowel habits, all occurring without any identifiable structural abnormalities. Understanding IBS is crucial for effective management and improving the quality of life for those affected [40]. The Rome VI Criteria classifies four different IBS patient subgroups based on their bowel habits and stool consistency, assessed using the Bristol Stool Scale [40,41].

This multifactorial disorder affects approximately 5% to 11% of the worldwide population across all age and sex groups, with a higher prevalence in females [42]. IBS symptoms must last at least three months and start no more than six months before the diagnosis [40]. The symptoms can significantly impact an individual’s quality of life, leading to discomfort and disruption in daily activities [42]. In the diagnosis of this functional disorder, it is important, in addition to the correct identification of the symptoms, to exclude suspicious organic pathologies characterized by the same symptoms [40]. Currently, there are no molecular biomarkers available to confirm the clinical diagnosis.

#### 2.2.1. IBS Etiopathogenetic Mechanisms

Despite the widespread prevalence of irritable bowel syndrome (IBS), the exact cause remains unknown. Research into its pathogenesis is ongoing, and several factors have been identified as potential contributors. Currently, we primarily have treatment strategies focused on managing symptoms and improving overall well-being [43,44] (Figure 1). Patients suffering from functional disorders of the gastrointestinal tract have altered visceral sensitivity and motor function in response to various external stimuli [45]. Furthermore, several external exposome factors, including diet and lifestyle, play a decisive role in altering the composition of the intestinal microbiota, influencing the symptoms’ persistence [45]. Indeed, the immune response due to bacterial translocation can lead to inflammation in the gut mucosa and exacerbate symptoms [46]. Furthermore, systemic immune activation contributes to IBS extra-intestinal symptom development such as fatigue, joint pain, and mood disturbances [47]. For these reasons, it is important to consider that distinct etiopathogenetic mechanisms may be responsible for various subtypes of IBS. Understanding these differences can lead to more targeted and effective treatment strategies, as summarized in Table 2 [41,43].

#### 2.2.2. Epigenetic Factors

Genetic factors modestly contribute to the development of irritable bowel syndrome (IBS); despite this, it is important to highlight the importance of understanding our hereditary influences on health. There is mounting evidence that environmental factors play a significant role, with numerous studies highlighting a greater incidence of stressful experiences among individuals with IBS. These include early adverse or traumatic events during childhood and ongoing stressors in adulthood [5]. The epigenetic changes influence gene expression without altering the underlying DNA sequence and can lead to changes in phenotype, contributing to the development or exacerbation of IBS symptoms [5].

Recent studies are exploring the interaction between the microbiome, diet, and epigenetics, known as the “microbiota–nutrient metabolism–epigenetics axis”, in complex diseases. Evidence suggests that epigenetic changes are dynamic and responsive to changes in nutrient availability and the microbiome. While the specific role of microbes and their metabolites in epigenetic processes underlying IBS symptoms has not been fully investigated, there is indirect evidence suggesting their involvement [45]. These interactions may be mediated by metabolites synthesized by commensal bacteria, such as neurotransmitters or short-chain fatty acids (SCFAs), believed to play a crucial role in the brain–gut microbiome axis in IBS. SCFAs can inhibit histone deacetylases, promoting an active chromatin state and thereby enhancing transcription; this suggests a potential link between the gut microbiome, dietary factors, epigenetic modifications, and the manifestation of IBS symptoms [45].

Nutrigenomics, the study of how diet interacts with our genes, is gaining attention in understanding IBS. Many patients with IBS experience symptoms related to meals, suggesting dietary changes as part of the treatment in their management [48]. For example, a low FODMAP diet, which reduces certain carbohydrates, has been linked to symptom relief in IBS [49]. Specific dietary components and vitamins, such as S-adenosyl methionine (SAM), folate, vitamin B12, vitamin B6, and acetyl-CoA, have been shown to regulate histone modifications or DNA methylation levels. This highlights the potential of diet to influence gene expression and contribute to IBS management.

#### 2.2.3. Diet and Intestinal Microbiota 

Changes in gut microbiota are linked to IBS and affect gut movement, immune response, and abdominal sensitivity, leading to diarrhea and stomach discomfort [50]. Understanding the role of intestinal bacteria and their interactions with the host epithelium and immune system is crucial for elucidating the pathogenesis of IBS and developing targeted therapeutic interventions. Strategies aimed at restoring microbial balance, improving epithelial integrity, and modulating immune responses may hold promise for managing IBS and alleviating its symptoms [51].

Elevated levels of Firmicutes and reduced *Bacteroides* were described in individuals with IBS compared to healthy volunteers. However, studies about microbiota composition in IBS showed conflicting results [52]. A recent study demonstrated that patients with IBS, particularly those with diarrhea-predominant and mixed subtypes, exhibited a different microbial profile compared to healthy controls. Specifically, they had a higher prevalence of the *Bacteroides* enterotype, while healthy controls showed a higher abundance of *Prevotella species* [53]. The study proposes that changes in the gut microbiota composition might lead to alterations in cytokine levels, triggering inflammatory responses, in particular certain bacteria in the microbiome, such as *Firmicutes*, producing flagellin, promotes an inflammatory response in individuals with IBS. This hypothesis is supported by findings showing significantly higher serum levels of lipopolysaccharide in patients with IBS-D compared to controls, as well as higher levels of antibodies to flagellin compared to controls [53]. Moreover, the current literature suggests that dietary differences among individuals may influence variations in the diets of patients with IBS, potentially impacting their gut microbiota composition [54,55].

#### 2.2.4. Environmental Factors 

IBS is influenced by various environmental factors, including chronic stress experienced during early life or adulthood, dietary habits, and gastrointestinal infections. Chronic stress can not only increase an individual’s susceptibility to developing IBS but can also trigger or worsen the symptoms associated with it [42].

Stress, but also depression and anxiety, disrupts the body’s homeostasis and has effects on gut physiology. It can lead to changes in intestinal motility, mucosal transport, and gut barrier function, resulting in alterations in permeability and visceral perception. These physiological responses to stress are primarily mediated by the sympathetic nervous system and the corticotropin-releasing factor axis pathways [45]. Glucocorticoids, including cortisol and corticosterone, play crucial roles in the hypothalamic–pituitary–adrenal axis’s response to stress. However, chronic and uncontrollable stressors can lead to maladaptive changes in stress response systems, ultimately affecting the structure and function of the brain. These maladaptive changes may contribute to the development or exacerbation of symptoms associated with IBS. The bidirectional relationship between the brain and the gut suggests that emotional states can influence gastrointestinal function and vice versa. Understanding the intricate relationship between stress and IBS can inform therapeutic interventions aimed at managing both the psychological and physiological aspects of the condition.

Recent studies have shown that altered intestinal barrier permeability may contribute to the pathophysiology of IBS [56]. Normally, this barrier regulates the passage of substances between the gut lumen and the bloodstream. Increased intestinal permeability can lead to the translocation of harmful substances and activate immune responses, contributing to gastrointestinal symptoms and inflammation [51].

Air pollution, consisting of gasses and particulate matter (PM), including PM2.5 (particles with a diameter < 2.5 µm), exerts a significant impact on health. PM can infiltrate the respiratory system and be ingested with food, reaching the gastrointestinal tract [57]. Once absorbed, certain particles can translocate into the bloodstream, eliciting an immune response. It is estimated that a Western diet may lead to the daily ingestion of 10^12^–10^14^ PM particles [58]. Exposure to these particles has been associated with systemic inflammation and oxidative damage to the colonic mucosa, thereby elevating the risk of chronic gastrointestinal disorders such as IBS even if no data are currently available about this connection.

## 3. miRNAs as Regulators of Hallmarks of IBD and IBS

Given the lack of specific diagnostic biomarkers for IBD and IBS, our goal is to provide a comprehensive list of miRNAs associated with these diseases from the existing literature. We aim to identify miRNAs that could serve as reliable diagnostic biomarkers. To achieve this, we utilized bioinformatics tools to systematically analyze relevant studies and gather information on miRNAs that are crucial in regulating key biological processes related to IBD and IBS.

We identified several miRNAs that regulate the expression of important mRNAs, which are crucial in the regulatory processes essential for the development of IBD and IBS. These miRNAs play a role in influencing disease-related characteristics and may also contribute to the onset of cancer [13,14,15]. We focused on those miRNAs with a role in IBD/IBS associated with the inflammatory and proliferative processes, immune system recruitment, oncogenesis, and intestinal epithelial barrier integrity [13,59,60,61]. In this review, we will also discuss how miRNAs influence the immune system by regulating the differentiation and function of immune cells, such as T cells, B cells, macrophages, and dendritic cells exacerbating or mitigating this response [62,63].

Moreover, considering that the integrity of the intestinal epithelial barrier is crucial for preventing gut inflammation, we will highlight the dysregulation of miRNAs involved in controlling intestinal permeability.

## 4. The Approach: Bibliographic Research and Data Extraction

### 4.1. Automated Data Retrieval from PubMed

A pipeline was developed in R to query bibliographic information from PubMed and organize it into a user-friendly database. The pipeline automates the collection of information and ensures that the data are accurate and up to date. To achieve this, several R packages were used, including “RISmed” to define the query, “easyPubMed” to extract the data, and “rentrez” to interact with the PubMed API. These tools were essential in obtaining precise and detailed information from research articles available on PubMed. The query used for this research was built directly from the PubMed database to identify relevant studies on the diseases of interest, namely inflammatory bowel disease, which includes UC, CD and IBS. The keywords “miRNA” and “microRNA” in the title or abstract were included to identify studies focused on these genetic elements. The period considered for publication was from 2019 to the present (1 May 2024). Lastly, all studies except reviews, systematic reviews, and conferences were considered to focus on original studies and primary research.

The query is defined as follows:

*‘(colitis[Title/Abstract] or crohn disease[Title/Abstract] or inflammatory bowel disease[Title/Abstract] or irritable bowel syndrome[Title/Abstract]) and (english[Language]) and (mirna[Title/Abstract] or microrna[Title/Abstract]) and (“2019/01/01”[Date* *–Publication]: “3000”[Date* *–Publication]) NOT (“review”[Publication Type]) NOT (“systematic review”[Publication Type]) NOT (“congress”[Publication Type])’*

**Figure 2:**
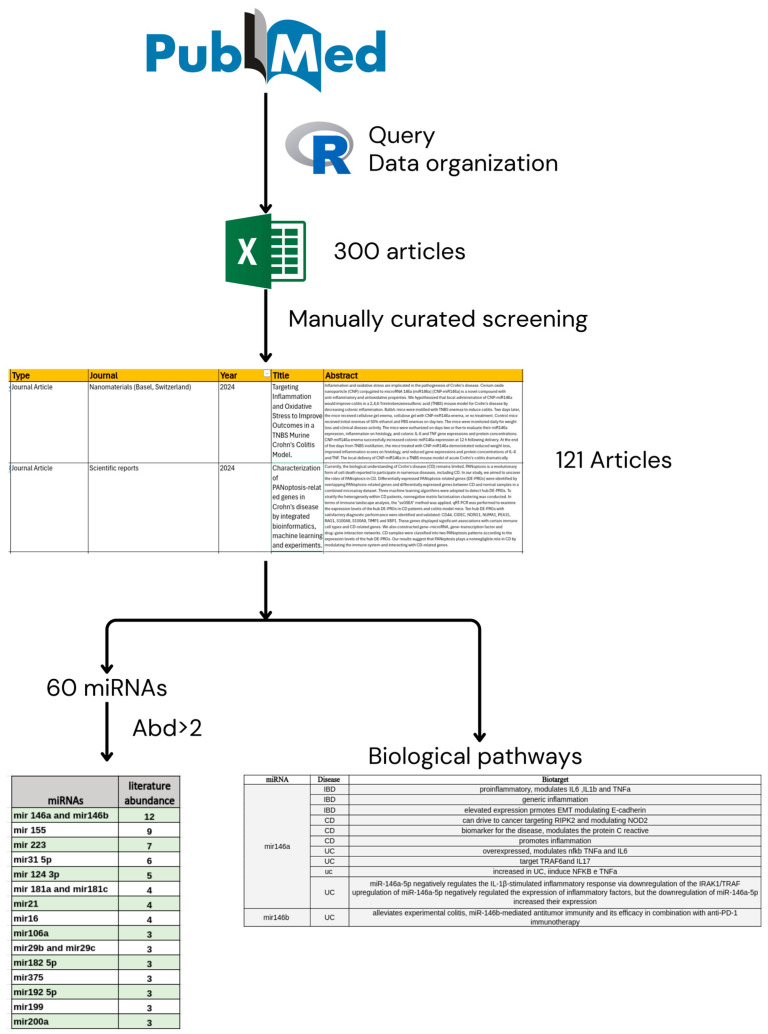
Graphical representation of the investigation scheme. An initial set of 300 articles on IBS and IBD was automatically retrieved from the PUBMED database. We then manually selected 121 articles, excluding those that were off-topic, were overly generic in terms of miRNA analysis/assay, or had a low impact factor. From the miRNAs mentioned in the articles, we focused on those studied in more than three articles (Abd > 2). The outcome of this workflow includes two tables: one listing the selected miRNAs and the other detailing the associated biological pathways.

In the R pipeline, the search query was implemented to retrieve the PMIDs (PubMed Identifiers) of the articles meeting these criteria. Subsequently, the abstracts of the retrieved articles were extracted and divided into two batches to avoid limitations imposed by the PubMed API, which restricts the number of articles that can be returned in a single query to prevent system overload. The extracted information for each article includes the article’s title and abstract (which provides an initial indication of the study’s content), the publication year of the article (to evaluate the recency of the research), the type of publication (to distinguish between original articles, clinical studies, etc.), the name of the scientific journal in which the article was published, and the keywords associated with each article (to facilitate categorization and thematic research). All this information was organized into a data frame, a structured data format that allows for easy manipulation and analysis of the data. In total, 300 articles were obtained (Figure 2), which were subsequently subjected to a manual editing and curation process to ensure the quality and relevance of the collected information.

### 4.2. Manually Curated Extraction of miRNA and Biological Mechanisms

Based on the initial selection of the previous database of 300 papers, and after the exclusion of any retracted articles, in the subsequent screening phase, we scanned each document by checking the abstract and, if necessary, the full text to see if the document was relevant for our research study.

To do this, we used the following exclusion criteria. First, we considered articles that were more frequently cited and/or with an impact factor of 4 or higher, which may reflect a greater level of influence and academic recognition within the field. Then, we considered the biological meaning of the study, the relative inherence of the topic, and the study’s strengths, which could impact the reliability and generalizability of the findings (this means the exclusion of papers in which the study was too generalized or those using organism models or targets incompatible with our study aim). We considered, at least, if the number and quality of the “Materials and methods” described in the selected articles were detailed enough to include them in the list.

A total of 121 articles were finally selected from this screening for subsequent analysis to identify specific miRNAs associated with CD, UC, IBD, or IBS. Our primary aim in this review was to define reliable miRNAs able to classify IBD from IBS for their possible use as diagnostic biomarkers. Subsequently, we aimed to correlate them to specific hallmarks of IBD and IBS and to disease-associated mechanisms that need to be investigated in order to better understand the molecular details of these pathologies. To this end, we identified 60 miRNAs mentioned in the articles as crucially related to IBD/IBS. From these, we selected 18 miRNAs that were studied in more than three articles to establish a strong and clear connection to the literature. Of note, we identified 18 miRNAs among these, several key ones that are frequently studied by research groups, including *miR-16, miR-21, miR-29b/c, miR-31-5p, miR-106a, miR-124-3p, miR-146a/b, miR-155, miR-181a/c, miR-182-5p, miR-192-5p, miR-199, miR-200a, miR-223,* and *miR-375*, and those that could be defined as the master regulators of hallmarks of IBD and IBS; additionally, we found other miRNAs critically involved but still not widely studied. Therefore, we categorized five complex biological mechanisms significantly related to IBD or IBS as IBD/IBS hallmarks: 1. inflammation or immune recruitment, 2. apoptosis or cell proliferation, 3. increased oxidative stress, 4. induced cell motility or tissue permeability, and 5. oncogenesis. We describe these hallmarks related to IBD and IBS and clarify the molecular pathways regulated by these different miRNAs.

### 4.3. Technical Approaches and Methodologies Used in the Study of IBD and IBS Disease

In this review, we reported a consistent number of manuscripts (121) that reported several different approaches and detection methods that reflect a wide variability of data sensibility and also their final aims. In particular, we observed that the gold standard for the study of IBS and IBD is the mouse or rat model, in which dysbiosis is induced by dextran sodium sulfate (DSS), lipopolysaccharide (LPS), 2,4,6-trinitrobenzene sulfonic (TNBS), or acetic acid treatment; many other studies were conducted starting with patient biopsies, tissue microarray, or blood/serum sample profiling compared to healthy patients. Secondary models were also in vitro colon cell lines or organoid models, engineered by silencing or ectopic expression of miRNAs or treated with vesicles or nanoparticles; other study models are based on the metanalysis model, where the bioinformatic approach was used to compare the open access GEO datasets.

In those models, most of the time transcriptomic analysis was conducted by RNA-seq or qPCR after acid nucleic extraction from biopsies, tissue, or blood samples supported by target-specific enzyme-linked immunosorbent assay (ELISA), immunohistochemistry (IHC), and immunogenetics proteomic assays, such as Western blot assay or luciferase assays. The articles that showed the combination of several methodologic approaches on different experimental models, especially for those with the same miRNA target, are limited in number. Furthermore, this review also reported single-miRNA-based articles, investigated by a few methods, made reliable due to the high-quality approach of the methods used (such as a combination of transcriptomic, proteomic, or other -omics assays) or the large numbers of the analyzed samples (such as a patient cohort study or comparative database investigation).

## 5. IBD and IBS Hallmarks: The Regulatory Role of microRNAs

### 5.1. Inflammation and Immune Recruitment

The IBD pathogenesis (including CD and UC) involves an interplay between genetic, environmental, and microbial factors. Immune system dysregulation and increased inflammation are the main issues in this group of diseases [1,64]. Therefore, understanding these mechanisms is crucial for developing targeted therapies to manage and treat these pathologies effectively [65,66,67,68]. As mentioned, there are genetic and environmental factors contributing to the development of IBD such as a high-fat diet, smoking, and life stress but also NOD2 gene mutations, which are associated with immune susceptibility to IBD. Environmental factors, such as infections and antibiotic use, can trigger or worsen IBD by affecting the gut microbiota [66,69]. Nevertheless, immunological factors play a crucial role in these diseases. Dysregulation of both the innate and adaptive immune systems leads to an inappropriate immune response against intestinal microbiota and cytokine imbalance; in turn, an overproduction of pro-inflammatory cytokines and reduced anti-inflammatory cytokines contribute to chronic inflammation in IBD [70,71]

The cytokine imbalance is a crucial element for the disease arising; indeed, an overproduction of pro-inflammatory cytokines (e.g., TNF-α, IL-6, IL-12, IL-23) and a reduction in anti-inflammatory cytokines (e.g., IL-4, IL-10, IL-11, TGF-b, Foxp3, and IL-13) were observed. Furthermore, regulatory mechanisms fail to effectively counterbalance this inflammation. Several factors could initiate and sustain inflammation, such as gut dysbiosis, due to altered microbial imbalance triggering and perpetuating inflammation [72,73]. In addition, it was reported that there is a disruption of the antigen-presenting cells (APCs), like dendritic cells or macrophages, that present these antigens to T cells. Moreover, the disruption in the epithelial barrier, which allows luminal antigens to penetrate the mucosa, induces T-cell activation with increased production of cytokines like IFN-γ and IL-17 in dendritic cells or Th2 response with IL-5 and IL-13 [74,75].

Ultimately, these pathologies often lead to chronic inflammation, persistent immune activation, tissue damage, and fibrosis, which can lead to the formation of pseudo polyps and an increased risk of colorectal cancer [76,77,78].

Our bibliography analysis reported that many miRNAs are associated with inflammation or immune recruitment in patients with IBD: miR-223, miR-181, miR-124, miR-31, miR-106, and miR-146. Based on our analysis, mir223, one of the most globally studied in IBD, was found in seven papers to be overexpressed [79,80,81,82,83,84,85]. Salama et al. described how miR-223 regulates inflammation in IBD by controlling the TNFα/NFκB/NLRP3 inflammatory axis by exerting a counter-regulatory effect on NLRP3 expression. In addition, Swati Valmiki et al. documented how miR-125b and miR-223 contribute to inflammation by targeting the NFκB pathway [80,81]. Zhang et al. also reported that miR-223 improves intestinal inflammation by inhibiting IL-6/STAT3 signaling [82]. Moreover, Xin Chang et al. observed that macrophage-derived exosomal miR-223 acts as an intestinal barrier modulator by inhibiting TMIGD1 expression [85].

Another miRNA family described within the immunomodulator category in IBD pathologies is represented by miR-181. In our bioinformatic query, we found four papers describing the anti-inflammatory properties of miR-181a and miR-181c [86,87,88,89]. As described by Shen et al., this miRNA family drives macrophage polarization but also regulates dendritic cell recruitment by regulating ERK-MAPK signaling [86,87,88]. Therefore, even the miR-181 family could be used as an IBD biomarker.

MiR-124 is another important miRNA described as an inflammation regulator in patients with IBD. Our research found five articles about miRNA and patients with IBD [90,91,92,93] It was often found to be overexpressed; in particular, the Yang Luo group described this miRNA as an important regulator of IL6 expression by TNFa and STAT3 [92]. Furthermore, Huang et al. studied the role of miR-124-3p and observed that elevated miR-124-3p expression disrupts the colon mucus barrier and increases susceptibility to colitis by targeting T-synthase [91]. Interestingly, the mir124-3p role is also debated by Zhen Qin’s paper, which described the double nature of this miRNA, where nicotine protects against mouse Dextran sodium sulfate (DSS)-induced colitis by enhancing miR-124 expression, but an elevated expression of miR-124 in CD aggravates the disease [93].

Another miRNA directly involved in IBD, specifically in UC, is miR-31. We found this described in six related papers as upregulated in inflamed tissues [94,95,96]. In particular, miR-31 could modulate the immune system by the induction of regulatory T cells targeting various components involved in immune signaling pathways and influencing cytokine production [97,98]

In another study, the altered expression of miR-106 has been implicated in various immune and inflammatory pathways, potentially affecting the course of the disease [91,93].

**Figure 3:**
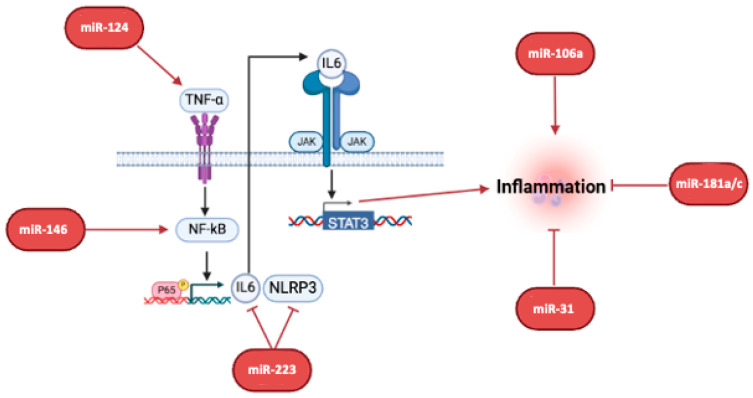
**Inflammation and immune recruitment.** The pathway mostly involved in the inflammation induced by miRNAs deregulation is TNF-a/IL-6/STAT3. Here, the main miRNAs involved in this process and how they contribute to IBD-IBS development are reported. Image created with Biorender (online version @2024).

MiR-146 has been thoroughly researched within IBD for its role in regulating the immune system; several papers reported an increase in miR-146a levels in the mucosa of patients with IBD. MiR-146a is involved in regulating inflammatory responses by targeting key signaling molecules such as the NF-κB pathway, with TRAF6, IL17, and IRAK1, and in STAT3/IL6 signaling, which are both crucial drivers for the activation of pro-inflammatory cytokines. Modulating these targets helps in the maintenance of immune balance, preventing excessive inflammation. Additionally, miR-146a is implicated in the differentiation and function of immune cells, including T cells and macrophages, which are significant in the pathogenesis of IBD. By influencing these cells, miR-146a can affect the overall immune response in the gut [99,100,101,102,103], as shown in Figure 3 and Table 3.

### 5.2. Apoptosis and Cell Proliferation

In IBD and IBS, the balance between proliferation and apoptosis is essential for maintaining the integrity of the intestinal epithelium; indeed, in IBD the regenerative ability of the intestinal epithelium is often compromised. Chronic inflammation leads to cycles of damage, where excessive cell proliferation tries to compensate for cell loss. Specifically, in UC, the crypts of Lieberkühn may exhibit increased proliferation in response to epithelial injury [159,160].

Among the molecular mechanisms described in the literature referring to IBD, we identified the ones related to growth factors such as EGF and TGF-β, which promote epithelial repair and proliferation [161,162]. In addition, in IBD, aberrations in the Wnt/β-catenin signaling pathway are crucial for cell proliferation; however, significant alterations in cell proliferation have not been reported [163].

Concerning the maintenance of cellular homeostasis, programmed cell death plays a distinct role in the context of IBD and IBS diseases [164,165]. In the IBS functional gastrointestinal disorder, the apoptosis role is still unclear, but it was barely connected to dysregulation and related to epithelial cell turnover and barrier functionality [166].

In IBD, compared to IBS, the dysregulation of apoptosis is quite well described; its imbalance (excessive or insufficient cell death) contributes to chronic inflammation and tissue damage. In UC, for example, epithelial cells in the colon may undergo excessive apoptosis, leading to ulceration and loss of the mucosal barrier [167]. Conversely, in CD, there is often resistance to apoptosis in activated T-cells, which perpetuates inflammation [168].

Indeed, the molecular mechanisms underlying apoptosis in IBD are closely linked to the overstimulated inflammatory state. The increased secretion of pro-inflammatory cytokines, such as TNF-α and IFN-γ, disrupts physiological tissue homeostasis and leads to dysregulated apoptotic processes [169]. Moreover, alterations in apoptosis-related genes including NOD2, ATG16L1, and IL23R further contribute to the disease’s pathogenesis [170]. Importantly, our bibliographic analyses highlighted several IBD/IBS-enriched miRNAs involved in the critical balance between proliferation and apoptosis.

Notably, miR-124-3p expression appears drastically downregulated in UC tissue. Luo et al. demonstrated that high levels of Rab27A enhance the STAT3/RelA signaling pathway by suppressing miR-124-3p, which in turn promotes apoptosis and contributes to the progression of UC [92]. Furthermore, the low expression of miR-192-5p in IBD correlates with a reduced capability to protect against intestinal injury induced by activated leukocyte cell adhesion molecule (ALCAM)-mediated inflammation and damage to intestinal epithelial cells. It was demonstrated that miRNA-192-5p inhibitors increased IL-1β and IL-6 levels and promoted IEC-6 cell apoptosis. On the contrary, miRNA192-5p overexpression induces cell viability and sustains reduction in the inflammatory response [155].

**Figure 4:**
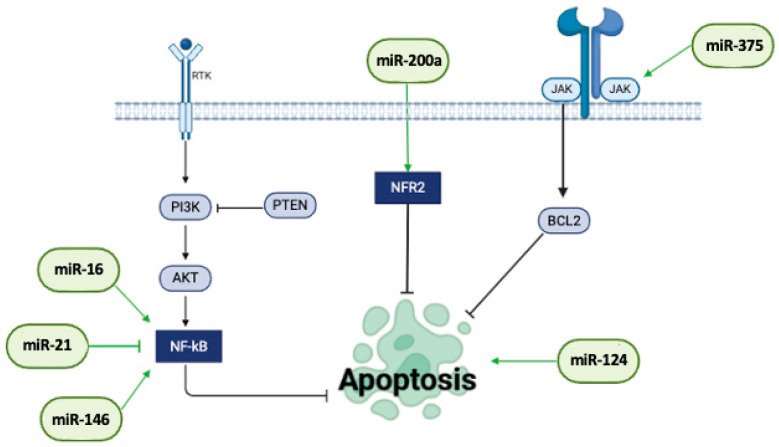
**Apoptosis and cell proliferation.** The pathway mostly involved in apoptosis induction is the PI3K/AKT axis; miRNAs also act on BCL2 gene expression, which has a pivotal role in anti-apoptotic processes. Here, the main miRNAs involved in this process and how they contribute to IBD-IBS development are reported. Image created with Biorender (online version @2024).

Similarly, miR-375 is downregulated in IBD and associated with the inflammatory environment typical of the disease. It was demonstrated that decreased miR-375 levels reduce human intestinal epithelial cell function viability and proliferation and induce apoptosis via the JAK2 axis [153]. Additionally, Chen et al. also showed that miR-375 downregulation in UC was associated with increased IRF7 levels and with SLC11A2 transcription inhibition. Importantly, the restoration of the IRF7/miR-375-3p/SLC11A2 axis alleviated pathological damage, decreasing ferroptosis and promoting proliferation [154] (Figure 4).

Other typically downregulated miRNAs involved in IBD apoptotic commitment are miR-200a, miR-146a, and miR-16. Peng et al. demonstrated that the high rate of apoptotic cells observed in in vivo DSS-induced colitis was restored after miR-200a overexpression. Coherently, Western blot analyses showed increased Bcl-2 expression correlated to a reduction in Bax protein amount [141]. Furthermore, the levels of miR-146a decrease proportionally with the severity of UC, being significantly lower in patients with severe forms of the disease compared to those with mild/moderate cases. MiR-146a knockdown inhibited cell apoptosis and inflammation by targeting TAB1 and suppressing the NF-κB pathway [102]. miR-16 is downregulated in patients with IBS with diarrhea and in mouse and cell models. The overexpression of miR-16 improves cell viability, reduces apoptosis and inflammation, and maintains intestinal tight junction integrity by targeting the TLR4/NF-κB signaling pathway [171]. Furthermore, Ye Chen MS et al. showed that high levels of miR-16 decreased expression of the anti-apoptotic protein Bcl-2 in the intestinal mucosa [172]. On the contrary, our analyses show that miR-21-5p results are typically upregulated in the sera and colon tissue of patients with UC compared to healthy controls [173]. Importantly, the downregulation of this miRNA inhibits apoptosis in UC in vitro models via the IL-6/STAT3 pathway, downregulating IL-6, TNF-α, IL6R, STAT3, ICAM-1, NF-κB, cleaved caspase-3, cleaved caspase-9, and FasL [174].

Finally, our findings underline that dysregulation of miRNAs expression significantly regulates the critical imbalance between proliferation and apoptosis in IBD and IBS. These molecular alterations could promote or inhibit cell death and proliferation in response to the inflammatory environment or tissue damage, affecting the severity of clinical manifestations. The key role of miRNAs in the pathogenesis of these gastrointestinal disorders highlights the importance of understanding miRNA-mediated mechanisms as potential biomarkers for disease development, as shown in Figure 4 and Table 3.

### 5.3. Oxidative Stress

Oxidative stress plays a critical role in the development of IBD and IBS, although through different mechanisms, and this should be considered a consequence of inflammation, which contributes to disease progression and complications such as fibrosis and cancer.

Both diseases reveal high inflammation and the activation of immune cells such as neutrophils and macrophages, and these cells produce reactive oxygen species (ROS) at high levels, leading to damage to epithelial cells and cellular structures, such as lipids, proteins, and DNA, ultimately damaging the lining of the gastrointestinal tract, disrupting the mucosal barrier.

In addition, those conditions allow luminal antigens and bacteria to penetrate into deeper tissues, perpetuating the inflammatory response.

Furthermore, patients with IBD often have reduced levels of antioxidants, such as superoxide dismutase and glutathione.

Our research identified several miRNAs implicated in the modulation of oxidative stress in IBD such as MiR-124, which suppresses apoptosis and induces ROS production, activating the STAT3 signaling pathway [92]; by contrast, miR-93a is a ROS modulator regulating NOX4 [175], but also miR-222-3p seems to be a crucial regulator of oxidative stress [46].

**Figure 5:**
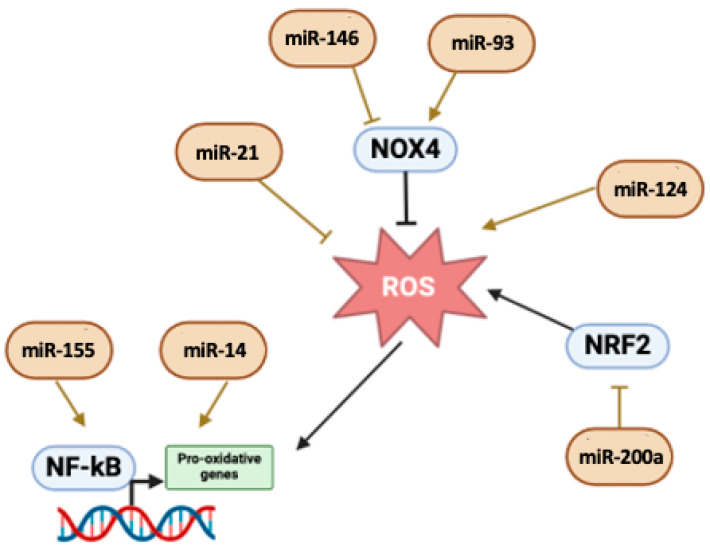
**Oxidative Stress.** NRF2, NOX4, and NFkB are among the proteins mostly involved in oxidative stress modulation. Here, the main miRNAs involved in this process and how they contribute to IBD-IBS development are reported. Image created with Biorender (online version @2024).

Other miRNAs seem to mitigate oxidative stress, such as miR-200a, reducing inflammation by the modulation of NRF2 [141]. Several other miRNAs, such as miR-155 and miR-21, were upregulated in both UC and CD and have been associated with the regulation of inflammatory responses and oxidative stress, with the first modulating the transcriptional activity of NF-κB [171] and the second impairing the antioxidant response of PTEN [46]. Moreover, miR-146a, as cited before, strongly regulates immune response and inflammation and targets the signaling pathways of key inflammatory mediators like TNF-α and IL-1β, which are also linked to oxidative stress [106,109,176]. Therefore, the interplay between microRNAs (miRNAs) and free radicals in inflammatory bowel disease (IBD) and irritable bowel syndrome (IBS) represents a crucial aspect of their pathogenesis and progression. This relationship revolves around how miRNAs regulate oxidative stress and inflammation and how free radicals, in turn, influence the expression and function of miRNAs. These small molecules modulate the main pathways involved in oxidative stress, inflammation, and tissue repair. As described, the balance between pro-oxidative miRNAs and anti-oxidative miRNAs determines the promotion or inhibition of oxidative stress and/or wide tissue inflammation. In addition, ROS presence can influence the expression of specific miRNAs by activating stress-related signaling pathways, creating a feedback loop that amplifies inflammation and tissue damage in IBD. In IBS, the miRNA-free radical interplay is less studied but still relevant. Subtle increases in ROS levels, mediated by dysregulated miRNAs, may contribute to altered gut motility and visceral hypersensitivity. Targeting specific miRNAs could modulate oxidative stress and inflammation, offering new avenues for treatment, such as inhibiting pro-oxidative miRNAs like miR-124 or miR-222-3p and enhancing the expression of anti-oxidative miRNAs such as miR-93a and miR-200a. [46,92,141,175] Therapies aimed at reducing ROS levels or enhancing antioxidant capacity could disrupt the feedback loop between free radicals and miRNAs, alleviating disease progression, as indicated in Figure 5 and Table 3.

### 5.4. Cell Motility and Tissue Permeability in IBD and IBS

Cell motility and permeability are increased in IBD and IBS due to an inflammatory response, which attracts immune cells like macrophages and neutrophils to the intestinal mucosa [177,178]. The breakdown of tight junctions between epithelial cells, which normally act as barriers, leads to a significant increase in tissue permeability, often called “leaky gut”, allowing bacteria and toxins to penetrate into deeper tissues, exacerbating inflammation. Cytokines and other inflammatory mediators also play a role in disrupting the epithelial barrier. IBS is also associated with increased gut permeability but is generally less pronounced. The increased permeability in IBS may result from mild inflammation, stress, or changes in gut microbiota, although the mechanisms are not as well understood as in IBD [179,180,181]. MiRNAs can modulate the expression of genes involved in the cytoskeleton and cell adhesion and influence the migration of immune and epithelial cells by affecting the inflammatory response and tissue repair processes. Moreover, miRNAs significantly impact the integrity of the intestinal barrier by modulating the expression of tight junction proteins and inflammatory mediators [182,183] or changes in gut microbiota, although the mechanisms are not as well understood as in IBD [179,180,181]. MiRNAs can modulate the expression of genes involved in the cytoskeleton and cell adhesion and influence the migration of immune and epithelial cells by affecting the inflammatory response and tissue repair processes. Moreover, miRNAs significantly impact the integrity of the intestinal barrier by modulating the expression of tight junction proteins and inflammatory mediators [182,183].

Our analysis revealed that three miRNAs are the master regulator of this focus: miR-155, miR-146a, and miR-223. As already reported, miR-155 is upregulated in IBD and is involved in immune cell activation and inflammation. It promotes the migration of immune cells like macrophages and T cells to the sites of inflammation by regulating the expression of chemokines and their receptors. Also, it influences cell motility and permeability, affecting epithelial cells by promoting the expression of inflammatory cytokines, which disrupt tight junctions and increase intestinal permeability [101,184,185,186,187,188]. miR-146a has regulatory effects on inflammation and immune responses; as already described, it targets key signaling molecules involved in inflammatory pathways, such as TRAF6, IRAK1, TNF-α, and IL-6 [101]. Therefore, its function in regulating the permeability of the barrier is given by its great regulatory effect on the inflammatory system. miR-223 is found at higher levels in the inflamed tissues of patients with IBD, and it plays a role in regulating the function of neutrophils and inflammation. It affects the movement and permeability of cells, influencing the recruitment and activation of neutrophils, and modulates the inflammatory responses that affect the integrity of the epithelial barrier.

**Figure 6:**
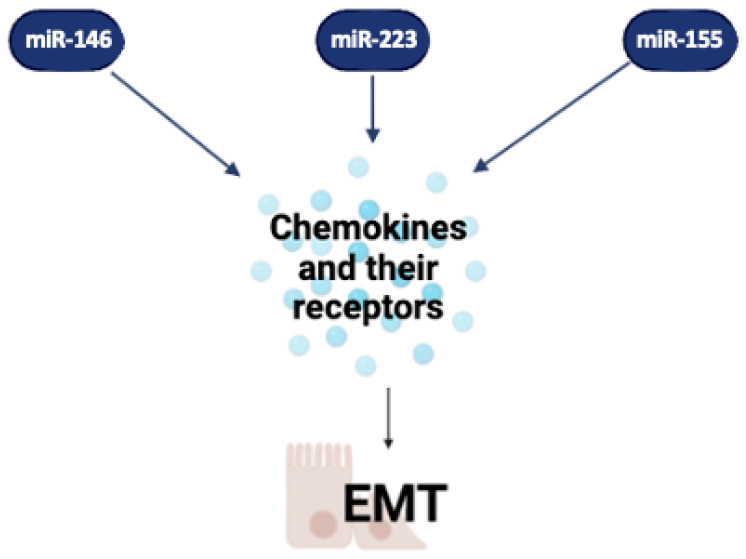
**Cell mobility and tissue permeability.** Here, we report three miRNAs among those mostly involved in chemokine production, which in turn contributes to gut epithelial barrier permeability disruption. Image created with Biorender (online version @2024).

In conclusion, understanding the specific roles of different miRNAs in these processes could provide insights into the pathogenesis of IBD and IBS and potentially lead to novel therapeutic approaches (Figure 6 and Table 3).

### 5.5. Oncogenesis

The risk of cancer developing is significantly high in patients with IBD, particularly in cases of UC, as chronic inflammation typically associated with IBD predisposes an individual to colorectal cancer [189,190].

Patients with IBD often have genetic predispositions that increase their susceptibility to both inflammation and tumorigenesis [191,192,193]. The accumulation of gene mutations, such as in NOD2 and p53, and the presence of epigenetic alterations raise the risk of oncogenic transformations. Genetic perturbations are entwined with the persistent inflammatory conditions of these disorders, which in turn contribute to causing DNA damage, promoting mutations, and affecting the normal regulatory mechanisms of cell growth and death [77,194,195].

Additionally, cancer development in IBD is often associated with alterations in the gut microbiome.

According to Refs. [196,197,198], some bacterial species could produce carcinogenic metabolites or promote inflammation, contributing to the oncogenic process [76,199,200].

IBD-related inflammation could compromise the intestinal barrier, allowing more toxins and microbes to enter the mucosa and potentially lead to oncogenic changes [201,202]

Of note, accumulating data indicates that IBD-associated colorectal cancer (CRC)(IBD-CRC) may initiate and develop through a pathway of tumorigenesis distinct from that of sporadic CRC [190,201,203].

Here, our bibliographic analyses highlight several miRNAs potentially involved in regulating oncogenic processes in the context of IBD and IBS disorders.

One of the most reported upregulated miRNAs in inflammatory disorders with oncogenic potential is miR-21. It promotes cell proliferation, angiogenesis, and the invasiveness of malignant cells [204,205]. Interestingly, colon tissue samples derived from patients with CD and UC exhibit significantly high levels of miR-21 compared to non-IBD controls [206]. Lai et al. demonstrated in a zebrafish model that miR-21 could trigger CRC or colitis-associated cancer by activating PI3K/AKT, STAT3, and PDCD4/TNF-α signaling pathways. It exacerbates the inflammatory response, disrupts the balance between tumor suppressor and oncogene expression, and accelerates tumorigenesis [207].

MiR-146a is a further key player associated with the risk of oncogenesis in IBD. In myeloid cells, it strongly modulates IL-17-mediated responses by targeting RIPK2, a NOD2 signaling intermediate, thus suppressing IL-17-induced cytokine production. The deletion of miR-146a in myeloid cells promotes CRC development. Additionally, in intestinal epithelial cells, miRNA-146a limits the response to IL-17 by targeting TRAF6 and further suppresses CRC by inhibiting PTGES2, an enzyme involved in PGE2 synthesis [105].

Furthermore, the deletion of miR-146b affects tumor progression in IBD. It reshapes and increases the tumor-associated macrophage (TAM) population and enhances macrophage reprograming to exert protumor activity and to impair immunosuppressive responses. Importantly, high levels of miR146b have been shown to alleviate colitis, promoting antitumor immunity in vivo. This effect is improved with the combination of anti-PD-1 immunotherapy [109]. 

Then, it was found that miR-155 is strongly involved in IBD tumorigenesis. Its high levels in primary sclerosing cholangitis with UC induce microsatellite instability and promote STAT-3 expression via SOCS1 inhibition. Moreover, miR-155 overexpression inhibits deficient mismatch repair (MMR) proteins and modulates p53, promoting neoplastic transformations [110]. Recently, SOCS1 has been reported to be involved in the degeneration of ulcerative colitis to tumoral tissues, and the regulatory network is represented by hsa-let-7d-5p, hsa-miR-16-5p, hsa-miR-145-5p, hsa-miR-223-5p, and hsa-miR-331-3p [208]. These miRNAs are associated with a poor prognosis of CRC. Also, miR-346 has been recently described with oncogenic properties in patients with PSC-UC. It modulates the expression of receptor of vitamin D (VDR) and TFNα, regulators of carcinogenic processes. Thus, the upregulation of miRNA-346 in PSC may lead to inadequate suppression of neoplastic stimuli [209].

Instead, the downregulation of miR-222-3p has been correlated with an ameliorated UC condition. This miRNA plays a protective role against colitis-associated colorectal cancer development, targeting the Nrf2/HO-1 signaling pathway, thereby reducing the inflammatory and oxidative responses of the tissues [46].

**Figure 7:**
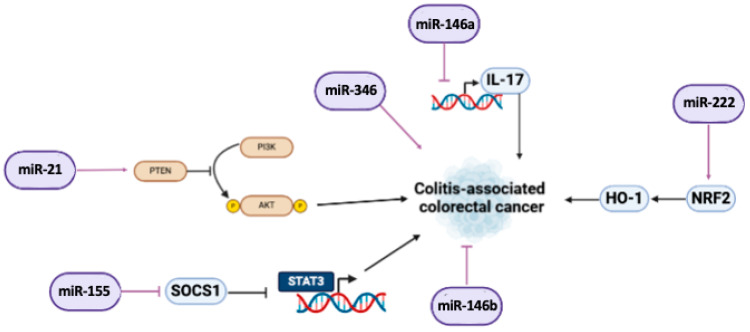
**Oncogenesis.** The pathways mostly involved in colorectal cancer (CRC) rise are PI3K/AKT axis and STAT3 and its downstream genes. Moreover, there is a modulation of interleukin accumulation and hypoxic state promotion. Here, the main miRNAs involved in this process and how they contribute to CRC onset are reported. Image created with Biorender (online version @2024).

The molecular mechanisms driving the pathogenesis of IBD underline the importance of investigating the key players involved in their clinical complications. Our findings focus on the significant role that miRNAs play in the severity and progression of these gastrointestinal disorders. Chronic inflammation, oxidative stress, and the disruption of microenvironmental homeostasis predispose these disorders to malignant transformation, in which altered miRNA expression may act as a trigger or modulator of oncogenesis (Figure 7 and Table 3).

## 6. Conclusions and Clinical Impact

This review would like to highlight the potential of miRNAs as IBD and IBS biomarkers for the early diagnosis and the management of these disorders. Identifying biomarkers capable of diagnosing the pathology in a simple, rapid, and non-invasive way, and also to be able to classify the pathology, could have a decisive role in future clinical practice. MiRNAs have been described in recent studies as biomarkers with theranostic potential. Growing evidence supports the significant role of genetic factors in these diseases due to specific gene mutations involved in immune responses and autophagy or due to epigenetic factors, including DNA methylation and histone modification. A critical role in these pathologies has been attributed to the composition of the microbiota. In addition, environmental factors influence their onset, such as diet, smoking, and geographic location. Despite the long list of scientific documents debating the physiopathology of these disorders, a critical point regarding the diagnosis is that there are no standardized tests, and current diagnostic practices rely on a combination of clinical, radiological, endoscopic, and histopathological evaluations. The common biomarkers used are fecal calprotectin and C-reactive protein, which should be considered helpful, but there is a pressing need for more specific biomarkers.

For all these reasons, meeting the needs encountered in clinical practice, this review aimed to collect diagnostic miRNAs, obtained by a bioinformatic selection of the IBD/IBS-related literature, that are suitable in the gastroenterological context. An R pipeline allowed us to select 300 papers, among which 120 articles passed the adopted inclusion criteria. These articles mentioned 59 miRNAs as having a role in IBD/IBS diseases, and these 59 miRNAs were studied in more than three articles. These miRNAs (miR-16, miR-21, miR-29b/c, miR-31-5p, miR-106a, miR-124-3p, miR-146a/b, miR-155, miR-181a/c, miR-182-5p, miR-192-5p, miR-199, miR-200a, miR-223, miR-375) were involved in the control of functional hallmarks of IBD and IBS, as increased inflammation and immune response recruitment/activation, apoptosis, and cell proliferation increased oxidative stress and induced immune cell mobility and epithelial tissue permeability and colorectal cancer onset. Considering that miRNAs could be referred to as the first molecules respondent to external stimuli and that miRNA-based therapies are being developed in several diseases [210], the identified miRNAs could also be considered as a potential target for the development of new therapeutic interventions and the management of chronic IBD and IBS disorders. Therefore, among all the cited miRNAs, some like miR-125b, miR-223, miR-181a, miR-124, and miR-31 were the most relevant in controlling inflammation and immune responses in IBD and IBS; they could be used to develop diagnostic tools and treatments for these diseases, defining a new molecular signature for diagnosis, optimal in terms of clinical costs/benefits. The possibility of isolating miRNAs in biological fluids underlines the importance of using them as a liquid biopsy, with a lower impact on the patient.

**Figure 8:**
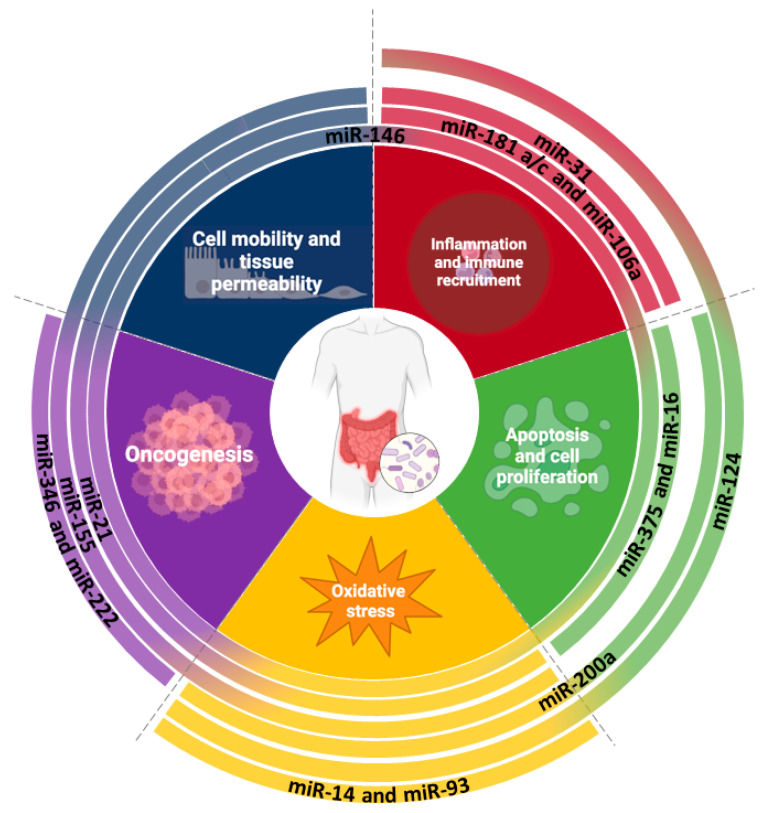
**miRNAs involved in IBD and IBS onset.** This image recapitulates the most cited miRNAs implicated in IBD and IBS development, and through which pathways they influence the pathology. Image created with Biorender (online version @2024).

In conclusion, understanding the interplay between genetic predisposition, environmental influences, and epigenetic mechanisms is essential for elucidating the complex pathophysiology of chronic gastrointestinal disorders (summarized in Figure 8). It highlights the importance of considering both genetic and environmental factors in the assessment and management of the condition, with potential implications for the development of targeted therapeutic interventions.

As a last consideration, we are aware that this study may have some limitations related to the used papers, such as the variability in miRNA expression among populations, the standardization of miRNA detection, and regulatory matters on clinical implementation. We note that the methods of standardization, the statistical power, and the choice of the cohort were used as the gold standard for screening the articles. In addition, the papers were reported to be methodologies and standardized protocols for sample collection, RNA extraction, and miRNA quantification, which can help reduce variability in the results. Moreover, when we considered a paper with data about patient samples, the cohorts were large and varied in order to obtain a representative spectrum of biological variability. Ultimately, incorporating various tissue types or distinct cohorts (such as different ethnicities, disease stages, or treatment groups) can aid in recognizing consistent miRNA signatures and addressing variability across diverse contexts.

Nevertheless, despite the above observed limitations, this study represents an optimal integration of clinical and basic research, highlighting a well-balanced approach between the two domains.

## Figures and Tables

**Table 1:** Clinical and pathological characteristics of IBDs.

	IBDs	
	CD	UC
Location	Entire digestive tract—mouth to anus The most affected areas are the small intestine, the terminal ileum, and the colon	Colon and rectum; normally does not spread beyond to ileum
Degree of inflammation	Transmural inflammation	Superficial mucosal inflammation
Histology	Granulomas, increased globet cells	Crypt abscesses, depletion of goblet cells
Smoking	Predisposing factor	Protective factor
Extraintestinal Manifestation	Erythema nodosum, pyoderma gangrenosum, arthritis, ocular manifestations	
Endoscopy	Skip lesions, deep ulcers, cobblestone appearance	Continuous lesions, pseudopolyps
Complications	Strictures, fissures, fistula, venous thromboembolism, colorectal cancer	Toxic megacolon, venous thromboembolism, increasing risk of colorectal cancer compared to CD
Associations	Gallstones	Primary sclerosing cholangitis (PSC)
Incidence	Approximately 25% of patients with IBD are under the age of 20, especially in adolescence; however, approximately 20% of children with IBD will present before 10 years of age, and approximately 5% will present before 5 years of age	The disease can arise at any age, but it most frequently appears between the ages of 20 and 40, with no gender predisposition

**Table 2:** Clinical characteristics of IBS subtypes and hypothesized etiopathogenetic factors.

Category	Clinical Characteristics	Possible Etiopathogenesis
IBS	Chronic abdominal pain Altered bowel habits (diarrhea, constipation, or both) Bloating Meal-related symptoms	Brain–gut axis dysfunction Gut dysbiosis Visceral hypersensitivity Altered intestinal motility
SUBTYPE		
IBS-D	Frequent diarrhea with loose or watery stools Possible mucus in stools Urgency and pain related to evacuation	Increased pro-inflammatory mediators Gut dysbiosis with fermentative bacteria Accelerated intestinal motility
IBS-C	Constipation with hard or lumpy stools Abdominal pain often not relieved by defecation Sensation of incomplete evacuation and straining	Slowed intestinal motility Increased colon sensitivity Increased intestinal permeability and altered gut microbiota
IBS-M	Mixture of constipation and diarrhea Frequent bloating and abdominal distension Symptom variability over time	Dysfunction of the brain–gut axis Altered gut microbiota Altered intestinal motility
IBS-U	Symptoms that do not fit a specific subtype Wide variability in symptom presentation Possible combination of IBS-D and IBS-C symptoms	Possible overlap of characteristics from other subtypes Undefined pathological mechanism Potential gut dysbiosis

**Table 3:** Summary of the main miRNAs involved in IBD/IBS and biological mechanisms. The upregulated miRNAs are indicated in red, and the downregulated miRNAs are indicated in blue.

miRNA	Disease	Functional Role in the Diseases	References
MiR-146a	IBD	It modulates IL6, IL1b, and TNFa expression and acts as a pro-inflammatory inducer. The elevated expression promotes EMT modulating E-cadherin.	[100,101,104]
	CD	It contributes to cancer by targeting RIPK2 or modulating NOD2 and C-reactive protein, leading to increased inflammation. It should be recognized as a potential biomarker for disease.	[89,103,105]
	UC	It modulates NF-κB, TNFα, and IL-6 while targeting TRAF6 and IL-17. It downregulates the IRAK1/TRAF pathway and negatively regulates the IL-1β-induced inflammatory response.	[79,99,102,106,107,108]
MiR-146b	UC	It alleviates colitis, enhancing antitumor immunity, and its effectiveness is increased, combining anti-PD-1 immunotherapy.	[109]
MiR-155	IBD	Elevated expression promotes EMT by downregulating E-cadherin. It acts as a miRNA with pro-inflammatory activity, regulating FOXP3 in T regulatory cells and influencing CTLA-4.	[104,110,111,112,113,114,115]
	UC	MiRNA overexpression protects the intestinal barrier by modulating the ROCK1 pathway. Matrine and Lactobacillus paracasei treatment can inhibit the miRNA, reducing pro-inflammatory activity linked to obesity.	[104,116,117,118,119,120]
	IBS	MiRNA inhibition alleviates irritable bowel syndrome, increasing claudin-1 and ZO-1 expression.	[115,121,122]
MiR-223	IBD	It regulates TMIGD1 expression and contributes to intestinal barrier dysfunction exacerbation.	[84,85,123]
	CD	It was found to be overexpressed in CD patients; should be used as biomarker.	[124,125]
	UC	It is overexpressed in the disease, and some probiotic agents, like Lactobacillus paracasei, can downregulate miRNA expression. It regulates NLRP3 expressions. It is also involved in colorectal cancer.	[80,81,82,83,126]
MiR-31-5p	UC	miRNA regulates colitis by affecting CD4 T cells. GOS treatment also reduces colitis, impairing miRNA expression. The miRNA’s expression is influenced by IL-6 and TNF-a and is highly expressed in the disease, regulating cytokines like p65 and STAT3.	[94,95,118,127]
	UC/CD	This miRNA was found to be overexpressed in inflamed tissues of the patient.	[128]
MiR-124-3p	IBD	Modulates T synthetases.	[91,93,129]
	CD	It represents a biomarker for the disease.	[130]
	UC	Suppresses apoptosis and induces ROS production; activates the STAT3 signaling pathway.	[90,92,131]
MiR-21	CD	Overexpressed in the disease.	[132]
	IBD	Drives inflammation and should be considered a premetastatic biomarker.	[133,134,135]
MiR-181a/c	CD	Diagnostic biomarker.	[89]
	IBD	It modulates MAPK expression in dendritic cells.	[87,88]
	UC	It inhibits M1 macrophage polarization and promotes the activation of M2, reducing inflammation.	[86,88,136]
	IBS	It decreases inflammation.	[137]
MiR-16	UC	Influences disease development.	[132,138,139]
	IBD	Its increased expression correlates with disease.	[132,138,139]
	IBS	It was found to be overexpressed in the microbiota.	[140]
MiR-200a	UC	It mitigates oxidative stress and reduces inflammation by the modulation of NRF2.	[141]
	IBD	It was found to be downregulated in the disease.	[142]
	CD	Upregulated in CD.	[128]
MiR-106a	UC	Influences UC development.	[127,143]
	IBD	This miRNA is overexpressed in patients with IBD and regulates IL-10/STAT3 signal transduction. It is an immune-suppressive miRNA, promoting T reg induction and suppressing anti-inflammatory cytokines.	[144,145]
MiR-29b/c	UC	It is downregulated when LIF is high. It promotes inflammation.	[146,147]
	CD	Highly expressed in patients with CD and modulates the tight junction by the decrease in PMP22 expression.	[148]
	IBS/IBD	Targets TRAF3 to regulate NF-κB-MLCK.	[149]
MiR-182 5p	UC	It promotes ulceritis by the WNT/Bcatenin and claudin-2 ways.	[150,151,152]
	UC/CD	Its inhibition prevents ulceritis and inactivates WNT/Bcatenin.	
MiR- 375	UC/CD	It regulates the SLC11A2 axis, promotes ferroptosis in colonic epithelial cells of patients with ulcerative colitis, induces upregulation of IRF7 and downregulation of JAK2 gene targets, and regulates SNHG5 to promote apoptosis. It is downregulated in the disease and regulates JAK2.	[79,125,153,154]
MiR-192-5p	UC	Biomarker for UC; induces inflammation by IL6 and IL1b.	[79,155,156]
	IBD	Reduces apoptosis; overexpressed.	[155]
MiR-199	CD/ UC	Overexpressed in patients.	[157]
	IBD	Overexpressed; useful as a biomarker. It represents a good restoring agent for UC interacting with map3k4, thereby suppressing pro-inflammatory MAPK and NF-κB signaling.	[112,158]

## Data Availability

Data sharing is not applicable to this article as no datasets were generated or analyzed during this current study.

## Abbreviations

IBD: inflammatory bowel disease; IBS: irritable bowel syndrome; miRNAs: micro ribonucleic acid; CD: Crohn’s disease; UC: ulcerative colitis; NOD-2: nucleotide oligomerization domain 2; ATG16L1: autophagy-related 16 like 1; IRGM: immunity-related GTPase M; UTR: untranslated region; CT: computed tomography; MRI: magnetic resonance imaging; CDAI: CD Activity Index; HBI: Harvey–Bradshaw Index; RPC-IPAA: restorative proctocolectomy with ileal pouch–anal anastomosis; ESR: erythrocyte sedimentation rate; DNA: deoxyribonucleic acid; CpG: cytosine followed by guanine; EWAS: early warning System for Cultural Heritage; IL-23R: interleukin 23 receptor; NF-kB: nuclear factor kappa B; GWAS: genome-wide association studies; IL-12B interleukin-12B, JAK2 Janus kinase; STAT3: signal transducer and activator of transcription 3; CCR6: C-C motif chemokine receptor 6; Th17: T helper 17 cells; WSD: Western-style diet; PUFAs: polyunsaturated fatty acids; SCFA: short-chain fatty acids; PAHS: polycyclic aromatic hydrocarbons; PM: fine particulate matter; SAM: S-adenosyl methionine; FODMAP: fermentable oligosaccharides, disaccharides, monosaccharides, and polyols; TNF-α: tumor necrosis factor-alpha; TGF-b: transforming growth factor, beta; Foxp3: forkhead box P3; IFN-γ: interferon gamma; DC: dendritic cell; NLRP3: NLR family pyrin domain containing 3; STAT3: signal transducer and activator of transcription 3; TRAF6: tumor necrosis factor receptor-associated factor 6; IRAK1: interleukin 1 receptor-associated kinase 1; Rab27A: member RAS oncogene family; ALCAM: activated leukocyte cell adhesion molecule; IRF7: interferon regulatory factor 7; SLC11A2: solute carrier family 11 member 2.
